# Supplementary material for: Organic carbon inventories in natural and restored Ecuadorian mangrove forests
Source: PeerJ. 2014 May 22;2:e388. doi: 10.7717/peerj.388 (PMC4034597; doi:10.7717/peerj.388)

Appendix 1. Plot characteristics and uncorrected carbon storage estimates (representing the average of two replicate cores wherever possible). Missing data indicate results which were highly uncertain (e.g. unable to obtain geographic coordinates, unable to penetrate sediment with coring device, entire core full of driftwood, etc.)

| Plot | Aboveground Biomass  (kg·Ha^-1^) | Tree Density (10^3^ trees·Ha^-1^) | Maximum Core Length (cm) | Sediment Carbon Storage (kg·m^-3^) | Distance to Estuarine Edge (m) |
| --- | --- | --- | --- | --- | --- |
| Nat A 1 | 126506 | 15408 | 83 | 29.86 | 70 |
| Nat A 2 | 91494 | 816 | 83 |  | 70 |
| Nat A 3 | 27973 | 17500 | 89 | 38.91 | 90 |
| Nat A 4 | 68137 | 13929 | 89 | 40.71 | 10 |
| Nat A 5 | 7407 | 612 | 89 | 19.03 | 10 |
| Nat A 6 | 37231 | 20000 | 83 | 39.03 | 30 |
| Nat B 1 | 191671 | 10064 | 89 | 30.26 | 50 |
| Nat B 2 | 88247 | 10204 | 71 | 32.64 | 260 |
| Nat B 3 | 385796 | 928 | 77 | 35.53 | 100 |
| Nat B 4 | 30204 | 2908 |  |  |  |
| Nat B 5 | 136228 | 816 | 89 | 48.3 | 40 |
| Nat B 6 | 165729 | 612 | 89 | 62.86 | 50 |
| Nat C 1 | 66118 | 408 | 95 | 43.7 | 310 |
| Nat C 2 | 13112 | 612 | 77 | 34.51 | 110 |
| Nat C 3 | 56981 | 2564 | 89 | 41.42 | 140 |
| Nat C 4 | 44263 | 408 | 71 | 32.49 | 110 |
| Nat C 5 | 13000 | 600 |  |  |  |
| Nat C 6 | 127711 | 80 | 53 | 30.82 | 420 |
| Rest A 1 | 20478 | 7500 | 89 | 49.47 | 230 |
| Rest A 2 | 16115 | 22500 | 71 | 59.94 | 100 |
| Rest A 3 | 38491 | 12500 | 83 | 60.8 | 200 |
| Rest A 4 | 37321 | 10000 | 35 | 15.21 | 230 |
| Rest A 5 | 19423 | 20000 | 59 | 29.14 | 230 |
| Rest A 6 | 33161 | 35000 | 95 | 38.57 | 90 |
| Rest A 7 | 12452 | 7500 | 83 | 41.0 | 140 |
| Rest B 1 | 78615 | 5204 | 95 | 46.25 | 10 |
| Rest B 2 | 20000 | 59932 | 89 | 41.74 | 30 |
| Rest B 3 | 26394 | 15000 |  |  |  |
| Rest B 4 | 27342 | 3316 | 47 | 33.43 | 50 |
| Rest B 5 | 38431 | 27500 | 83 | 56.83 | 70 |
| Rest B 6 | 28392 | 62500 |  |  |  |
| Aff 1 | 109189 | 5816 | 89 | 39.58 | 110 |
| Aff 2 | 56845 | 5612 | 83 | 49.32 | 110 |
| Aff 3 | 21311 | 3316 | 89 | 34.92 | 110 |
| Aff 4 | 63847 | 3316 | 65 | 35.26 | 230 |
| Aff 5 | 30796 | 3112 | 89 | 55.08 | 190 |
| Aff 6 | 277984 | 7908 | 89 | 55.01 | 150 |

Appendix 2. Total aboveground biomass is correlated with patch location. All sites follow the equation biomass = -0.139(distance) + 65.5, with a confounding effect of 142.1 for site Nat B.


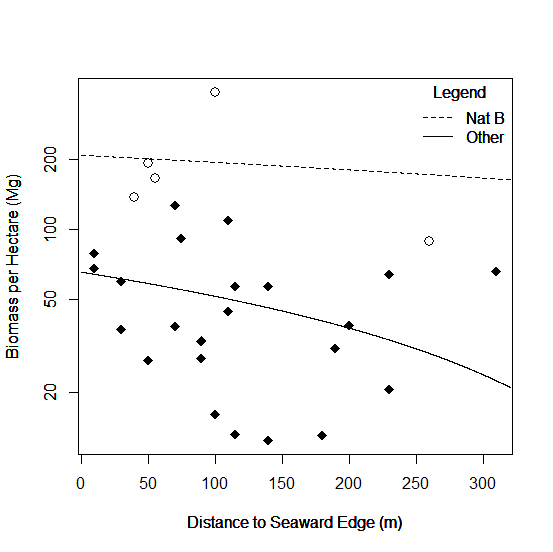

Supplement: Supplemental Information 1 — The paper by Hackerott et al (2013) claims to find no effect of grouper on lionfish abundance in the Caribbean Appendices for DelVecchia et al. “Organic carbon inventories in natural and restored Ecuadorian mangrove forests”. [file peerj-02-388-s001.docx]
